# The value of cognitive behavioral therapy on quality of life in addition to pharmacotherapy in adults with ADHD

**DOI:** 10.1192/j.eurpsy.2022.1564

**Published:** 2022-09-01

**Authors:** R. Wettstein, Y. Klabbers, E. Romijn, J. Nieuwveen, H. Kroesen, K. Wettstein, G. Dumont

**Affiliations:** 1 Amsterdam university medical center, Clinical Pharmacology And Pharmacy, Amsterdam, Netherlands; 2 ADHDcentraal, Research & Development, Nieuwegein, Netherlands; 3 GGNet, Ivb, Warnsveld, Netherlands

**Keywords:** Cognitive behavioral therapy, Quality of Life, adults, adhd

## Abstract

**Introduction:**

Treatment options for ADHD in adults consist of psycho-education, cognitive behavioral therapy (CBT), pharmacotherapy or a combination thereof. Current studies do not yet provide insights into the additive effects of CBT and pharmacotherapy regarding the quality of life in adults with ADHD.

**Objectives:**

In this study, we investigated the effect of CBT combined with pharmacotherapy on the quality of life in adults with ADHD compared to pharmacotherapy alone.

**Methods:**

In this multicenter prospective cohort study a total of 627 patients were included, 305 where included in the pharmacotherapy only group and 322 in de combination group (CBT and pharmacotherapy). The Adult ADHD Quality-of-Life scale (AAQoL) was conducted at baseline and at the end of treatment.

**Results:**

No significant differences were found in gender or age between groups at baseline. The average improvement in the AAQoL total score in the pharmacotherapy group was 26.81(17.12) and in the combination group 25.45(16.33) and showed no significant difference (t(543) = 0.96, *p* = 0.34). At baseline the average total score in the pharmacotherapy group was 45.5(12.37) and 42.22(12.73) in the combination group (t(543)=2.86, *p* = 0.004). The average total score at the end of treatment in the pharmacotherapy and combination group was 72.31(12.99) and 67.67(12.45), respectively (t(543)=426, p <0.001).

**Conclusions:**

To our knowledge, this is the first study to describe the value of CBT in addition to pharmacotherapy on the quality of life in adults with ADHD. Contrary to our expectations, there was no significant effect of CBT in addition to pharmacotherapy on the quality of life.

**Disclosure:**

No significant relationships.

